# Risk Factors for Colorectal Cancer in Patients with Multiple Serrated Polyps: A Cross-Sectional Case Series from Genetics Clinics

**DOI:** 10.1371/journal.pone.0011636

**Published:** 2010-07-16

**Authors:** Daniel D. Buchanan, Kevin Sweet, Musa Drini, Mark A. Jenkins, Aung Ko Win, Dallas R. English, Michael D. Walsh, Mark Clendenning, Diane M. McKeone, Rhiannon J. Walters, Aedan Roberts, Sally-Ann Pearson, Erika Pavluk, John L. Hopper, Michael R. Gattas, Jack Goldblatt, Jill George, Graeme K. Suthers, Kerry D. Phillips, Sonja Woodall, Julie Arnold, Kathy Tucker, Amanda Muir, Michael Field, Sian Greening, Steven Gallinger, Renee Perrier, John A. Baron, John D. Potter, Robert Haile, Wendy Frankel, Albert de la Chapelle, Finlay Macrae, Christophe Rosty, Neal I. Walker, Susan Parry, Joanne P. Young

**Affiliations:** 1 Familial Cancer Laboratory, QIMR, Herston, Queensland, Australia; 2 School of Medicine, University of Queensland, Herston, Queensland, Australia; 3 Ohio State University, Columbus, Ohio, United States of America; 4 Gastroenterology and Hepatology Department, Canberra Hospital, Garran, Australian Capital Territory, Australia; 5 Centre for MEGA Epidemiology, School of Population Health, University of Melbourne, Carlton, Victoria, Australia; 6 Queensland Clinical Genetics Service, Royal Children's Hospital, Herston, Queensland, Australia; 7 School of Paediatrics and Child Health, University of Western Australia, Nedlands, Western Australia, Australia; 8 Genetic Services of Western Australia, Subiaco, Western Australia, Australia; 9 Department of Paediatrics, University of Adelaide, Adelaide, South Australia, Australia; 10 South Australian Clinical Genetics Service, North Adelaide, South Australia, Australia; 11 Familial GI Cancer Registry, Auckland City Hospital, Auckland, New Zealand; 12 Hereditary Cancer Clinic, Prince of Wales Hospital, Randwick, New South Wales, Australia; 13 Department of Clinical Genetics, Royal North Shore Hospital, Sydney, New South Wales, Australia; 14 Illawarra Cancer Centre, Wollongong Hospital, Wollongong, New South Wales, Australia; 15 Samuel Lunenfeld Research Institute, Mount Sinai Hospital, Toronto, Ontario, Canada; 16 D. Zane Cohen Digestive Diseases Clinical Research Centre, Mount Sinai Hospital, Toronto, Ontario, Canada; 17 Cancer Care Ontario, Toronto, Ontario, Canada; 18 University of British Columbia and BC Cancer Agency, Vancouver, British Columbia, Canada; 19 Department of Medicine, Dartmouth Medical School, Hanover, New Hampshire, United States of America; 20 Cancer Prevention Program, Fred Hutchinson Cancer Research Center, Seattle, Washington, United States of America; 21 USC/Norris Comprehensive Cancer Center, University of Southern California, Los Angeles, California, United States of America; 22 Department of Colorectal Medicine and Genetics, The Royal Melbourne Hospital, Parkville, Victoria, Australia; 23 Department of Molecular and Cellular Pathology, University of Queensland, Herston, Queensland, Australia; 24 Envoi Pathology, Herston, Queensland, Australia; 25 Department of Gastroenterology, Middlemore Hospital, Auckland, New Zealand; Health Canada, Canada

## Abstract

**Background:**

Patients with multiple serrated polyps are at an increased risk for developing colorectal cancer (CRC). Recent reports have linked cigarette smoking with the subset of CRC that develops from serrated polyps. The aim of this work therefore was to investigate the association between smoking and the risk of CRC in high-risk genetics clinic patients presenting with multiple serrated polyps.

**Methods and Findings:**

We identified 151 Caucasian individuals with multiple serrated polyps including at least 5 outside the rectum, and classified patients into non-smokers, current or former smokers at the time of initial diagnosis of polyposis. Cases were individuals with multiple serrated polyps who presented with CRC. Controls were individuals with multiple serrated polyps and no CRC. Multivariate logistic regression was performed to estimate associations between smoking and CRC with adjustment for age at first presentation, sex and co-existing traditional adenomas, a feature that has been consistently linked with CRC risk in patients with multiple serrated polyps. CRC was present in 56 (37%) individuals at presentation. Patients with at least one adenoma were 4 times more likely to present with CRC compared with patients without adenomas (OR = 4.09; 95%CI 1.27 to 13.14; P = 0.02). For females, the odds of CRC decreased by 90% in current smokers as compared to never smokers (OR = 0.10; 95%CI 0.02 to 0.47; P = 0.004) after adjusting for age and adenomas. For males, there was no relationship between current smoking and CRC. There was no statistical evidence of an association between former smoking and CRC for both sexes.

**Conclusion:**

A decreased odds for CRC was identified in females with multiple serrated polyps who currently smoke, independent of age and the presence of a traditional adenoma. Investigations into the biological basis for these observations could lead to non-smoking-related therapies being developed to decrease the risk of CRC and colectomy in these patients.

## Introduction

Familial non-syndromic colorectal cancer (CRC) constitutes one of the most difficult and diverse patient groups encountered in a genetics clinic, with no apparent germline mutation, an often-indeterminant mode of inheritance, and questions arising as to how to manage the probands, and how to identify which family members are also at risk for CRC. One such condition is hyperplastic polyposis syndrome (HPS), a colorectal polyposis of unknown etiology characterized by the development of multiple serrated polyps in the large intestine. Efforts to define the clinical boundary of HPS have been hampered by heterogeneity of phenotype, with a vast array of polyp numbers, sizes [1], histological subtypes [2], and varying distributions in the colon leading to a set of recognition criteria which are necessarily stringent but which may exclude a significant number of high-risk cases [3]. The clinical significance of HPS is that it is associated with an increased personal and familial risk of CRC [4], [5], [6], [7], and extra-colonic cancers in the wider family setting [8].

Some 10 years ago, Rashid *et al* suggested that there are *at least* two phenotypic subtypes of HPS. The first demonstrates numerous hyperplastic polyps which may or may not be large, and such patients have also been described in series by Williams et al [9] and Ferrandez et al [10]. An alternative phenotype of HPS demonstrates fewer polyps than that described above however includes a diversity of polyp types including common hyperplastic polyps, serrated adenomas, sessile serrated adenomas, traditional adenomas, and polyps with mixed elements [1], [11]. This second phenotype of HPS is reported to be more likely to have polyps with diameters exceeding 1cm, dysplastic changes, to involve the proximal colon and to be associated with the presence of CRC [6]. Despite this, estimation of CRC risk in individual patients with HPS remains problematical [12], [13]. That there are at least two forms of HPS has been also suggested by others [14]. The reasons for this phenotypic dichotomy are currently unknown but may involve genetic backgrounds and environmental modifiers.

One environmental modifier may be cigarette smoking. The relationship between serrated neoplasia and smoking has been examined in a number of population-based studies [15], and evidence for an association has emerged for both precursor and malignant lesions in the serrated pathway [16], [17], [18], [19], [20], [21]. Multiple independent studies have shown an identical pattern of higher risk estimates for serrated (hyperplastic) polyps than for adenomas [16], [17], [19] in long-term and current smokers. The highest risks of all were observed when both adenomas and serrated polyps were present [16], [17], [19]. In a meta-analysis of risk factors for serrated polyps, both common and advanced lesions were significantly associated with current cigarette smoking [22]. Consistent with this, several large population-based studies have demonstrated that the CRC subset bearing the molecular signature of serrated neoplasia, namely somatic *BRAF* mutation, CpG Island Methylator Phenotype (CIMP) and microsatellite instability (MSI) has the highest association with smoking [23] [18], [20]. Given these findings, the authors have explored the effects of cigarette smoking on phenotype and risk of CRC in a large series of high-risk patients with multiple serrated polyps attending genetics clinics. This study presented an opportunity to determine whether cigarette smoking contributed to the increased risk of CRC in these patients.

## Methods

This cross-sectional study comprised 151 Caucasian patients with multiple serrated polyps (with at least 5 occurring outside the rectum) recruited from genetics clinics in Australia and New Zealand (n = 139) and North America (n = 12) regardless of family history of polyps and cancer. This was done in order to target high-risk patients more likely to be predisposed to serrated polyps whilst simultaneously limiting the chances of recruiting patients with common late-onset distal serrated polyps. Forty-one patients from Australia were participants in the Colon Cancer Family Registry (Colon CFR) [24], 39 were from the Royal Melbourne Hospital Hyperplastic Polyposis Study [25], and the balance of participants were enrolled in the Genetics of Serrated Neoplasia (GSN) study (http://gsn.qimr.edu.au/index.html) through Cancer Care Ontario, Ohio State University Medical Center, the Combined Genetics Clinics of Australia and the Familial GI Cancer Registry of New Zealand. Thirty-six patients from Australia, and 8 from North America have been reported previously [25], [26]. Patients gave written informed consent to participate in research and the study was approved by the HREC of Queensland Institute of Medical Research under the Genetics of Serrated Neoplasia Project (QIMR HREC Protocol P912).

### Definitions

The use of the term *serrated polyp* in this report encompasses any polyp with serrated glandular architecture [27], and includes hyperplastic or metaplastic polyps, serrated adenoma, sessile serrated adenoma and mixed polyps. Minimum reported polyp count was obtainable from patient records in 120 individuals and in the remaining 31 individuals, polyposis was described as numerous (n = 2), prolific (n = 1) or multiple suggesting hyperplastic polyposis and prompting referral to a genetics clinic (n = 28). Polyposis was categorised into two groups where polyp count was known; moderate (5–79 polyps) and dense (≥80 polyps) [28], and into those fulfilling WHO HPS criterion 3 (>30 polyps throughout the colon) and those with polyp counts between 5 and 30 [3]. Cigarette smoking status of patients *at the time of initial diagnosis* with polyposis was categorised into 3 groups; never, former and current smokers. **Cases** were individuals with multiple serrated polyps who presented with at least one CRC at the time of initial diagnosis with polyposis. **Controls** were individuals with multiple serrated polyps who had not developed CRC at the time of initial diagnosis with polyposis. **Index** patients were those who presented independent of other family members, and screening cases were diagnosed subsequently to an index case in their respective families.

### Polyps and Cancers

Pathology review of polyps was undertaken by a specialist gastrointestinal pathologist (NIW). Twenty-eight CRC were available for analysis and underwent a *BRAF* V600E somatic mutation test as follows. The somatic T>A mutation at nucleotide 1799 causing the V600E mutation in the BRAF gene was determined using a fluorescent allele specific PCR assay. Briefly, 20–50ng of DNA, extracted from formalin-fixed paraffin-embedded tumour tissue, was amplified in a 25µl reaction containing 100nM each of allele specific primers tagged with differing fluorophores (Mutant Primer (F1): 6-Fam-5′-**CAGT**GATTTTGGTCTAGCTTCAGA-3′ Wild Type Primer (F2): NED - 5′- **T**GATTTTGGTCTAGCTACAGT-3′ and a common reverse primer (Reverse Primer (REV): 5′-CTCAATTCTTACCATCCACAAAATG-3′), together with 2.5units of Taq polymerase (Eppendorf), 1× buffer and 200µM of dNTPs. The cycling conditions consisted of an initial denaturation of 95°C for 2mins followed by 35 cycles of 94°C for 30sec, 59°C for 30sec and 65°C for 30sec then a final extension of 65°C for 10mins. After amplification 1µl of the PCR product was added to an 8.7µl mix of HiDi formamide and ROX Genescan 500 size marker (Applied Biosystems, Foster City, CA). The mutant allele (A1799) primer generated a PCR product of 97bp, 3bp larger than the wildtype PCR product after separation on an ABI 3100 genetic analyser. GeneMarker (SoftGenetics) software was used to identify the different size and fluorescent allele PCR products. Positive and negative controls were run in each experiment and 10% of samples were replicated with 100% concordance.

### Statistical Analysis

Logistic regressions were performed to estimate odds ratios (ORs) and 95% confidence intervals (95% CIs) for the associations between predictor variables and CRC. Patients with missing data were excluded from the analyses. To estimate the independent effect of cigarette smoking on the risk of CRC, we used multivariable logistic regression models adjusting for potential confounders including age at diagnosis, sex and presence of traditional adenomas. We also tested for interactions between sex and cigarette smoking status in association with CRC. The association between smoking and CRC was estimated separately for males and females. All regression models were compared using the Bayesian Information Criterion [29], [30].

All statistical tests were two-sided and *P*-value <0.05 was considered as a significant level of statistical evidence to reject the null hypothesis. All statistical analyses were done using Stata 10.0 [31].

## Results

In this analysis, a total of 151 patients diagnosed with multiple serrated polyps were recruited from Australia (n = 89), New Zealand (n = 50), Ohio, USA (n = 11) and Canada (n = 1). CRC was diagnosed in 57 (38%) individuals at presentation. The frequency of patients from Australasia presenting with CRC (37%) was not different significantly from that of North America (42%) (*P* = 0.8). Baseline characteristics for the study patients are shown in **Table 1**.

**Table 1:** Baseline characteristics of participants in the study.

	Cases (n = 56)	Controls (n = 95)
	n/N (%)	n/N (%)
Recruitment Site		
Australia	39/89 (44)	50/89 (56)
New Zealand	12/50 (24)	38/50 (76)
Ohio, USA	4/11 (36)	7/11 (64)
Canada	1/1 (100)	0/1(0)
Sex		
female	30/90 (33)	60/90 (67)
Age (years)		
mean (SD)	51.6 (15.0)	46.2 (14.6)
Adenoma		
No	4/27 (15)	23/27 (85)
Yes	48/109 (44)	61/109 (56)
Unknown status	4/15 (27)	11/15 (73)
Advanced serrated polyps		
No	15/41 (37)	26/41 (63)
Yes	22/58 (38)	36/58 (62)
Unknown status	19/52 (37)	33/542(63)
Minimum number of polyps reported		
mean (SD)	58 (55)	32 (24)
Polyposis		
moderate (5–79)	32/105 (30)	73/105 (70)
dense (≥80)	13/19 (68)	6/19 (32)
Unknown status	11/27 (41)	16/27 (59)
WHO Criterion 3		
No (5–30 hyperplastic polyps)	19/65 (29)	46/65 (71)
Yes (more than 30 hyperplastic polyps)	26/57 (46)	31/57 (54)
Unknown status	11/29 (38)	18/29 (62)
Cigarette smoking		
Never	26/61 (43)	35/61 (57)
Former	19/39 (49)	20/39 (51)
Current	10/49 (20)	39/49 (80)
Unknown status	1/2 (50)	1/2 (50)
Female Current Smokers	2/29 (7)	27/29 (93)
Male Current Smokers	8/20 (40)	12/20 (60)

a includes dysplastic serrated polyps.

SD = Standard Deviation.

Cases (n = 56) presented with CRC *and* polyposis, controls (n = 95) with polyposis only.

Of all participants, 33% (30/90) of female and 43% (26/61) of male patients presented with at least one CRC. Mean age at diagnosis of polyposis cases with CRC was 52 (standard deviation, SD 15) years ranging from 18 to 85 years while mean age at diagnosis of polyposis in controls (no CRC) was 46 (SD 15) years ranging from 16 to 76 years (*P* = 0.033). Where site was known, 62% (33/53) of CRC occurred in the proximal colon (*P*<0.001). Proximal cancer was less frequent in patients under 50 (8/21 or 38%) compared to patients aged 50 and over (25/32 or 78%) (OR = 0.17; 95%CI 0.05 to 0.58; *P* = 0.005). There was no significant difference between the sexes with respect to the location of the CRC (*P* = 0.48). Eleven of twenty eight CRC cases (39%) tested positive for somatic *BRAF* mutation and all 11 were located in the proximal colon. Patients presenting with CRC had significantly higher reported polyp counts (58, SD 55) than did those with no CRC (32, SD 24) (*P* = 0.002).

A total of 140 patients had *at least one* polyp reported as an adenoma. Of these, 49 (45%) individuals had CRC. Patients with colorectal adenomas were four times more likely to be diagnosed with CRC compared to patients without adenomas (OR = 4.09; 95%CI 1.27 to 13.14; *P* = 0.02) after adjusting for age, sex and smoking status (**Table 2**). Patients with dense polyposis (≥80 polyps) had 5 times increased odds of CRC compared to those with moderate polyposis (5–79 polyps) (OR = 4.31; 95%CI 1.74 to 16.24; *P* = 0.003). Similarly patients who met WHO HPS criterion 3 were three times more likely to have CRC compared to those who did not (OR = 2.63; 95%CI 1.15 to 6.00; *P* = 0.02). We found no statistical evidence for increased odds of CRC in patients with advanced serrated polyps (most of which were non-dysplastic sessile serrated adenomas) compared to those without (adjusted OR = 1.15; 95%CI 0.47 to 2.78; *P* = 0.76). All associations (ORs) were adjusted for age, sex and smoking status (not shown in the tables).

**Table 2:** Association between smoking, adenoma, sex, age and CRC in patients presenting with multiple serrated polyps.

	Univariate		Multivariate*	
	OR (95%CI)	*P*-value	OR (95%CI)	*P*-value
Cigarette smoking				
Never	1.00 (Referent)		1.00 (Referent)	
Former	1.28 (0.57 to 2.87)	0.550	0.71 (0.29 to 1.77)	0.463
Current	0.35 (0.15 to 0.82)	0.015	0.35 (0.14 to 0.88)	0.026
Never	1.00 (Referent)		1.00 (Referent)	
Ever#	0.67 (0.34 to 1.32)	0.247	0.50 (0.24 to 1.07)	0.075
Adenoma				
No	1.00 (Referent)		1.00 (Referent)	
Yes	4.52 (1.47 to 13.97)	0.009	4.09 (1.27 to 13.14)	0.018
Sex				
Female	1.00 (Referent)		1.00 (Referent)	
Male	1.49 (0.76 to 2.90)	0.247	1.57 (0.73 to 3.36)	0.245
Age (year)	1.03 (1.00 to 1.05)	0.033	1.01 (0.98 to 1.04)	0.510

*adjusted for other variables in the table.

# both former or current smokers.

Of patients diagnosed with CRC, 26 (47%) patients had no history of smoking while 19 (34%) and 10 (20%) patients were former and current smokers respectively. Odds of CRC for former smokers was not statistically different compared to never smokers (OR = 0.71; 95%CI 0.29 to 1.77; *P* = 0.46) after adjusting for age, sex and adenomas. In addition, there was no statistical difference in the average pack-year exposure between patients with and without CRC (mean pack-years 13.9 vs 14; *P* = 0.95). We found no significant differences in polyp counts between current smokers and former smokers for either males (mean polyp count 49 vs 41; *P* = 0.46) or females (41 vs 45; *P* = 0.74), and between current smokers and never smokers for either males (mean polyp count 49 vs 38; *P* = 0.36) or females (41 vs 37; *P* = 0.84).

**Figure 1:**
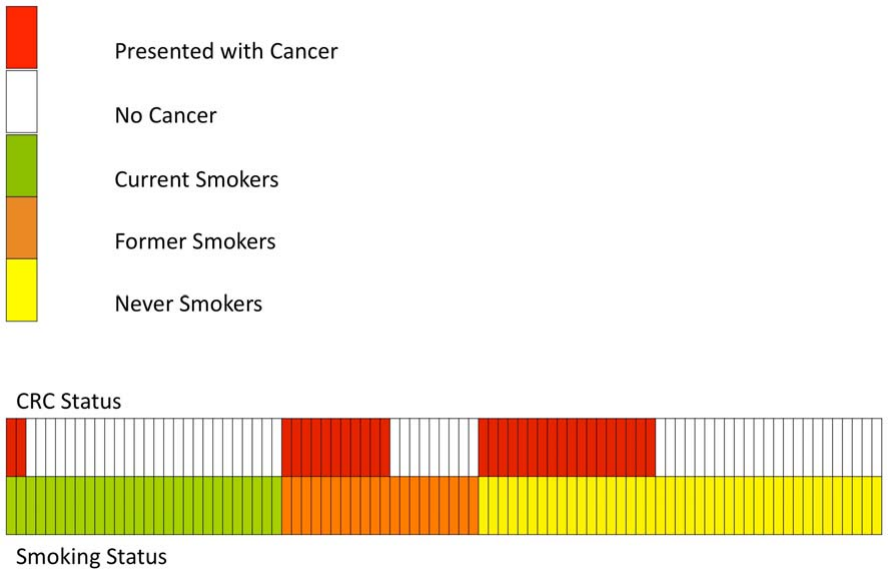
Schematic diagram of all female patients in the study with CRC and smoking status, showing the decreased odds of presenting with CRC in those currently smoking.

Current smokers were at 65% decreased odds of CRC compared with never smokers (OR = 0.35; 95%CI 0.14 to 0.88; *P* = 0.03) after adjusting for age, sex and adenomas. For females, the odds of CRC decreased by 90% in current smokers compared to never smokers (OR = 0.10; 95%CI 0.02 to 0.47; *P* = 0.004) after adjusting for age and adenomas. For males, there was no statistical evidence for an association between current smoking and CRC (**Table 3**). The association between current smoking and CRC for male and female HPS cases was statistically different (Interaction between current smoking and sex; *P* = 0.02). Of all patients in the study, 113/151 (75%) were index patients (those representing the initial diagnosis in each family). When the analysis was confined to index patients, the same pattern of decreased risk in females was observed (OR = 0.11; 95%CI 0.02 to 0.62; *P* = 0.01). A schematic diagram of the relationship between smoking status at diagnosis and presenting with a CRC is shown in **Figure 1**, and details of all female patients in the study are given in **Table S1**.

**Table 3:** Association between cigarette smoking and CRC in multiple serrated polyp patients stratified by sex.

	Females		Males	
Cigarette smoking	OR (95%CI)*	*P*-value	OR (95%CI)*	*P*-value
Never	1.00 (Referent)		1.00 (Referent)	
Former	0.85 (0.25 to 2.86)	0.795	1.00 (0.23 to 4.42)	0.996
Current	0.10 (0.02 to 0.47)	0.004	1.24 (0.26 to 5.86)	0.784
Never	1.00 (Referent)		1.00 (Referent)	
Ever#	0.31 (0.11 to 0.86)	0.025	1.18 (0.33 to 4.20)	0.799

*adjusted for age and adenomas.

# both former or current smokers.

## Discussion

In this cross-sectional series of high-risk patients with multiple serrated polyps, we have shown that the risk of CRC is increased in the presence of a synchronous adenoma, and decreased in females who are current smokers, regardless of age. In contrast, a positive association between smoking and colorectal neoplasia has been consistently reported. Multiple primary reports and meta-analyses have demonstrated that long term and current exposure to cigarette smoke is significantly associated with both colorectal cancer and its precursor lesions [16], [18], [19], [20], [21], [22], [32], [33], [34], [35], [36], [37], [38], [39], [40], [41], [42], [43], [44], [45], [46], [47], [48]. The evidence regarding precursor lesions is particularly strong. In a meta-analysis of 42 independent studies, smoking was significantly associated with adenomatous polyps with an OR of 2.14 (95%CI 1.86–2.46). Risk estimates were higher in studies where the control population had undergone colonoscopy [34], thus supporting an earlier hypothesis proposed by Terry and Neugut [49] that controls need to be screened in order that a more accurate risk estimate be obtained. When serrated polyps are examined, higher risk estimates than for adenomas are obtained, and higher again are the risk estimates from patients with both serrated polyps *and* adenomas [16], [17], [19].

When the association between smoking and colorectal cancer is analysed, risk estimates are decreased compared to those obtained when studying polyps, a phenomenon known as *the smoking paradox*. Two reasons for this have been proposed. Firstly that the effect of smoking on polyps may be greatest in small lesions with little malignant potential, and secondly that a longer latency period than the duration of many cohort studies may be required for smoking to exert its effects [34]. Whilst both of these reasons are likely to contribute to the smoking paradox, recent work on molecular sub-typing has demonstrated that smoking is significantly associated with the subset of colorectal cancers which harbor a somatic mutation in *BRAF* [18], and this may also contribute to the apparent dilution effect of smoking on colorectal cancer as *BRAF*-mutated cancers comprise only 10% overall. Where CRC were linked to somatic *BRAF* mutation, the risk was associated with long-term smoking [18].

Despite this evidence, international health-governing bodies such as the office of the US Surgeon General and the International Agency for Research on Cancer (IARC) have deemed this evidence insufficient to establish causality [32]. The major reason for this is that the results may be confounded by factors associated with both smoking and colorectal cancer such as physical activity [50], alcohol consumption [51], and diet [52]. A recent report however addressed both multiple risk factors and long duration of smoking in studying a large cohort of patients where the median duration of smoking was 44 years. The results confirmed that long-term smoking presented the highest risk for colorectal cancer, even after adjusting for multiple covariates known to affect risk [32].

Given the consistent findings from multiple reports that current smoking increases the risk of serrated polyps, and the significant association between smoking and colorectal cancer which develops via the serrated pathway, we studied a group of high-risk patients predisposed to develop multiple serrated polyps with a view to exploring the influence of smoking on the increased CRC risk present in this group of patients. Our results suggest that in patients predisposed to developing multiple serrated polyps, there is no significant association between smoking and an increased risk of presentation with CRC. In a recently-reported analysis of risk factors for serrated polyps, current smokers showed significant increases in risk for both common and advanced serrated polyps particularly in the distal colon [22]. In our study, CRC were more likely to be proximal, suggesting that if the effect of smoking in patients with multiple serrated polyps is to increase the likelihood of left sided polyps, then our findings that smoking overall has no significant effect on CRC development is consistent with this, as left colon serrated polyps are *less* likely to undergo malignant conversion. Of interest, a previously reported association with distal CRC was confirmed in our study in patients aged under 50 at presentation [25], [28], [53].

An unexpected finding of our study was that current smoking in females reduced the risk of presenting with CRC when compared to never smokers. This result remained significant after adjusting for both age at presentation and adenomas, as patients with a CRC were older at presentation than patients without a malignant lesion, as well as the finding of an increased risk of CRC conferred by at least one traditional adenoma. The two female patients in our study who were currently smoking and who presented with CRC were later-onset, and were among the long-term smokers (>40 years) as well as those with the highest polyp counts (122 and 129 respectively, with pan-colonic polyposis but interestingly concentrated in the recto-sigmoid). In a very recent publication, Walker and colleagues presented a series of 32 patients, predominantly female, with hyperplastic polyposis where 9 currently smoking female patients were described. Of note, three of these 9 patients had CRC and all 3 had recto-sigmoid polyp counts >50 [54]. Therefore it is likely that the females with multiple serrated polyps whose disease is more proximally located are the targets of this observed effect. In addition, the effect on female current smokers, though significant, was not complete, and suggests only a *subset* with a particular genetic background or who are within a dose-response window, are responding to a component of cigarette smoke, and this fraction will vary among patient cohorts. Polyp counts were significantly higher in patients with CRC in our series, however variances were large thus suggesting an *overlapping* distribution of cancer risk, and highlighting the complex nature of this condition. The most important risk factor for CRC remained the presence of a co-existing adenomatous lesion [6], [28], [55], the nature of which is a current area of investigation [56]. Of the 4 patients with CRC in whom no traditional adenomas were reported, one demonstrated multiple serrated adenomas (dysplastic serrated polyps). The confirmation that the presence of dysplasia in individuals with multiple serrated polyps is a risk factor for developing CRC will signal to clinicians the need for increased vigilance if continuing colonoscopic surveillance in these individuals and may trigger a discussion regarding surgery if all polyps cannot be removed.

Patients with HPS as currently defined are at a significantly increased risk of presenting with CRC, and are likely to represent a subset of the population with a particular genetic background [57]. Two large studies of 77 and 126 patients respectively, suggest that the risk of CRC in HPS is approximately 30–40% [28], [58], and coupled with a high background of somatic *BRAF* mutation (30–40% of CRC arising in HPS have a somatic *BRAF* mutation) [59], such an elevated risk may simply serve to overwhelm any effect of smoking on *BRAF*-mutated CRC. In the current report, we found no evidence that current smoking was more likely to be associated with a *BRAF*-mutated CRC. Of interest, a recent publication has shown that the *BRAF*-smoking-CRC axis is essentially confined to males [60].

The apparent decrease in CRC risk for currently smoking females is consistent with a biological mechanism akin to that observed in patients with ulcerative colitis which may be operating in a *subset* of female patients with multiple serrated polyps. The anti-inflammatory effects of smoking in ulcerative colitis are anecdotally well known, and the risk to females of CRC in ulcerative colitis is significantly decreased with respect to males [61]. The results reported here suggest that inflammatory processes may be responsible for neoplastic progression in serrated neoplasia predisposition in a *subset* of female patients. A risk factor study for serrated polyps has demonstrated that aspirin use decreased the risk of advanced proximal polyps, lending indirect support to this finding [22]. Alternatively, unspecified sex-specific factors related to body mass index (BMI), or hormonal factors, as are seen in the protective effects of smoking on endometrial cancer may be confounding the results reported here. Since no BMI or hormonal data were available for these patients at the time of writing, it is not possible to test this hypothesis. Of interest, decreased risks from current smoking for ASPs have been reported previously from a series of serrated polyp patients with no co-existing CRC or adenomas [62], and this work alludes to possible overlapping mechanisms with the findings of our report. Though cases in that report were not partitioned by sex, another previous report has shown that advanced serrated polyps are more likely to be present in females [63].

The results of our study suggest that current smoking neither accounts for, nor exacerbates, the high risk of CRC in patients predisposed to multiple serrated polyps. Further, in females with multiple serrated polyps, current smoking appears to be associated with a decreased risk of presenting with CRC. Due to the limited numbers in our analysis, our findings may be due to chance. The results of our study may also be influenced by ascertainment status of the patients, but when we reanalyzed our data using only index patients, the results remained significant. Three-quarters of our patients represented the first presentation in their respective families, and 70% of these had no affected relatives with CRC so were referred to the genetics service on the basis of having polyposis. A further effect on the outcomes reported here is that confounders including diet, obesity, physical activity, hormonal status and alcohol intake, may have influenced the results, and further larger studies will be needed to confirm our findings both in high-risk clinics and in the population. Our findings are unlikely to be broadly applicable to sporadic CRC cases as patients with multiple serrated polyps from genetics clinics are likely to have a particular genetic background, however, there may be some overlapping effects [62]. The direct effects of sex hormones alone can be ruled out by the number of females, many of whom are below the age of menopause, who were former or never smokers and who have presented with CRC. The increased proportion of patients with numerous serrated polyps who are current smokers has been noted previously [28], [54], and this observation requires further investigation to determine whether cigarette smoking enhances the phenotype [28], thus bringing it to clinical attention, or is associated with symptom relief in patients with a high polyp burden who therefore continue to smoke.

Importantly, investigations into the biological mechanism for our observation of decreased risk of CRC in females may lead to a CRC-preventive modality for female patients with serrated polyps *independent* of cigarette smoking and its attendant health risks, and may ultimately lead to a desirable reduction in the incidence of colectomy in the management of high-risk female patients with multiple serrated polyps. In addition, increased surveillance may be required in females when smoking cessation occurs to counteract any potential rebound effect. If this biological mechanism is an anti-inflammatory process, alternative therapies to nicotine could potentially be established in the treatment regimen of female patients with multiple serrated polyps.

## Supporting Information

Table S1 Details for all female participants with multiple serrated polyps. FH CRC = family history of CRC; ASP = advanced serrated polyp; AD = adenomas; CRC = presented with CRC at initial diagnosis; y = yes, n = no, u = unknown
(0.21 MB DOC)
