# Supplementary material for: Risk Factors for Colorectal Cancer in Patients with Multiple Serrated Polyps: A Cross-Sectional Case Series from Genetics Clinics
Source: PLoS One. 2010 Jul 16;5(7):e11636. doi: 10.1371/journal.pone.0011636 (PMC2905435; doi:10.1371/journal.pone.0011636)
Supplement: Table S1 — Details for all female participants with multiple serrated polyps. FH CRC = family history of CRC; ASP = advanced serrated polyp; AD = adenomas; CRC = presented with CRC at initial diagnosis; y = yes, n = no, u = unknown (0.21 MB DOC) [file pone.0011636.s001.doc]

| **Site** | **Index Patient Status** | **FH CRC** | **Age at Diagnosis** | **Polyp Number** | **ASP** | **AD** | **CRC** | **CRC Site** | **Smoking Status at Diagnosis** | **Pack Years** | **Exposure (years)** | **Cigarettes per Day** |
| --- | --- | --- | --- | --- | --- | --- | --- | --- | --- | --- | --- | --- |
| NZ | y | n | 59 | 122 | y | y | y | proximal | current | 40 | 40 | 20 |
| Aus | y | n | 64 | 129 | y | n | y | distal | current | 33 | 44 | 15 |
| NZ | y | n | 54 | 28 | y | y | n |  | current | 80.5 | 46 | 35 |
| Aus | y | n | 27 | 45 | y | y | n |  | current | 52.5 | 42 | 25 |
| NZ | n | y | 76 | 16 | n | y | n |  | current | 50 | 50 | 20 |
| Aus | y | y | 51 | 50 | u | y | n |  | current | 47 |  |  |
| Aus | y | y | 41 | 40 | u | n | n |  | current | 45 | 30 | 30 |
| Ohio | y | y | 60 | 30 | y | y | n |  | current | 45 | 45 | 20 |
| Aus | n |  | 44 | 5 | y | n | n |  | current | 36 | 36 | 20 |
| NZ | n | y | 42 | multiple | u | u | n |  | current | 33.75 | 27 | 25 |
| NZ | y | n | 53 | multiple | y | y | n |  | current | 33.3 | 37 | 18 |
| Aus | n | n | 48 | 74 | u | y | n |  | current | 33 | 33 | 20 |
| Ohio | y | n | 46 | 30 | y | y | n |  | current | 30 | 30 | 20 |
| Aus | y | y | 48 | 16 | n | y | n |  | current | 25 |  |  |
| NZ | n | y | 67 | multiple | u | u | n |  | current | 24 | 48 | 10 |
| Aus | y | n | 40 | 50 | n | y | n |  | current | 21 | 21 | 20 |
| Aus | y | y | 47 | 50 | y | y | n |  | current | 20 |  |  |
| Aus | y | n | 27 | 60 | u | y | n |  | current | 15 |  |  |
| NZ | y | y | 48 | 10 | n | n | n |  | current | 13.5 | 27 | 10 |
| NZ | y | n | 25 | 28 | y | n | n |  | current | 12.6 | 14 | 18 |
| NZ | n | y | 46 | multiple | y | n | n |  | current | 9.5 | 19 | 10 |
| Ohio | n | y | 56 | 7 | y | y | n |  | current | 8.75 | 35 | 5 |
| NZ | y | n | 31 | multiple | u | u | n |  | current | 7.5 | 15 | 10 |
| Aus | n | y | 50 | multiple | y | y | n |  | current | 7.5 | 30 | 5 |
| NZ | y | n | 64 | 13 | y | y | n |  | current | 6.9 | 46 | 3 |
| Aus | y | y | 17 | 75 | n | y | n |  | current | 6 | 8 | 15 |
| NZ | n | y | 27 | 30 | y | y | n |  | current | 4.5 | 9 | 10 |
| NZ | y | n | 17 | 13 | u | n | n |  | current | 1.75 | 5 | 7 |
| NZ | y | n | 65 | 150 | u | u | y | proximal | former | u | 3 | u |
| Aus | n | y | 67 | 40 | n | y | y | proximal | former | 50 |  |  |
| Aus | n | u | 63 | 30 | u | y | y | “colon” | former | 39 | 39 | 20 |
| NZ | y | n | 58 | 34 | y | y | y | distal | former | 25 | 25 | 20 |
| Aus | n | y | 48 | 45 | y | y | y | proximal | former | 24 | 32 | 15 |
| Ohio | y | y | 61 | 30 | n | y | y | proximal | former | 20 | 20 | 20 |
| NZ | y | n | 62 | 30 | u | u | y | proximal | former | 16 | 40 | 8 |
| Aus | y | y | 66 | multiple | u | y | y | “colon” | former | 8.6 | 43 | 4 |
| Aus | y | n | 42 | 50 | n | y | y | distal | former | 7 | 14 | 10 |
| Aus | y | n | 50 | 100 | u | y | y | proximal | former | 1 | 4 | 5 |
| Aus | y | y | 66 | 50 | n | y | y | proximal | former | 0.5 | 5 | 2 |
| Aus | y | n | 54 | 40 | y | y | n |  | former | 20 |  |  |
| Aus | y | y | 40 | 18 | u | n | n |  | former | 15 |  |  |
| Aus | y | y | 43 | 40 | y | y | n |  | former | 10 |  |  |
| Aus | y | y | 43 | 50 | y | y | n |  | former | 4 |  |  |
| Aus | y | y | 42 | 45 | n | y | n |  | former | 3 |  |  |
| NZ | y | n | 54 | 50 | n | y | n |  | former | 0.6 | 2 | 6 |
| NZ | n | y | 24 | 16 | n | n | n |  | former | 0.6 | 4 | 3 |
| Aus | n | y | 42 | 15 | y | y | n |  | former | 0.6 | 6 | 2 |
| NZ | y | n | 62 | 30 | n | y | n |  | former | 0.6 | 30 | 0.4 |
| NZ | y | n | 57 | prolific | y | y | y | distal | never | 0 | 0 | 0 |
| NZ | y | y | 51 | multiple | n | n | y | proximal | never | 0 | 0 | 0 |
| NZ | y | n | 41 | multiple | n | y | y | proximal | never | 0 | 0 | 0 |
| Aus | y | n | 47 | multiple | n | y | y | proximal | never | 0 | 0 | 0 |
| Aus | y | n | 38 | multiple | u | y | y | proximal | never | 0 | 0 | 0 |
| Aus | y | u | 85 | multiple | u | y | y | proximal | never | 0 | 0 | 0 |
| Aus | y | n | 23 | 300 | n | y | y | “colon” | never | 0 | 0 | 0 |
| Canada | y | y | 23 | 150 | y | y | y | distal | never | 0 | 0 | 0 |
| Aus | y | y | 47 | 50 | y | y | y | distal | never | 0 | 0 | 0 |
| Ohio | y | n | 19 | 50 | y | y | y | proximal | never | 0 | 0 | 0 |
| Aus | y | y | 35 | 30 | y | y | y | distal | never | 0 | 0 | 0 |
| NZ | y | y | 36 | 30 | n | n | y | distal | never | 0 | 0 | 0 |
| Aus | n | y | 61 | 30 | y | y | y | proximal | never | 0 | 0 | 0 |
| Aus | n | y | 76 | 30 | y | y | y | proximal | never | 0 | 0 | 0 |
| Aus | y | n | 78 | 15 | y | u | y | proximal | never | 0 | 0 | 0 |
| NZ | n | y | 58 | 15 | n | n | y | proximal | never | 0 | 0 | 0 |
| Ohio | n | y | 58 | 10 | y | y | y | proximal | never | 0 | 0 | 0 |
| Aus | y | u | 67 | 5 | u | y | y | proximal | never | 0 | 0 | 0 |
| Aus | y | y | 64 | 45 | n | y | u |  | never | 0 | 0 | 0 |
| NZ | y | y | 68 | multiple | u | y | n |  | never | 0 | 0 | 0 |
| NZ | y | y | 31 | multiple | u | u | n |  | never | 0 | 0 | 0 |
| NZ | y | n | 64 | multiple | n | y | n |  | never | 0 | 0 | 0 |
| NZ | y | y | 76 | multiple | n | y | n |  | never | 0 | 0 | 0 |
| NZ | n | y | 59 | multiple | y | n | n |  | never | 0 | 0 | 0 |
| NZ | n | y | 27 | multiple | y | y | n |  | never | 0 | 0 | 0 |
| Aus | n | y | 31 | multiple | y | n | n |  | never | 0 | 0 | 0 |
| Aus | y | y | 65 | 70 | y | y | n |  | never | 0 | 0 | 0 |
| Aus | y | y | 31 | 45 | u | n | n |  | never | 0 | 0 | 0 |
| NZ | y | n | 26 | 40 | y | y | n |  | never | 0 | 0 | 0 |
| Aus | y | y | 31 | 30 | u | y | n |  | never | 0 | 0 | 0 |
| Aus | y | y | 52 | 20 | u | n | n |  | never | 0 | 0 | 0 |
| Aus | y | y | 42 | 20 | u | n | n |  | never | 0 | 0 | 0 |
| Aus | n | y | 21 | 20 | u | y | n |  | never | 0 | 0 | 0 |
| Aus | y | y | 19 | 15 | y | y | n |  | never | 0 | 0 | 0 |
| Aus | n | y | 51 | 15 | u | y | n |  | never | 0 | 0 | 0 |
| Aus | n | y | 43 | 15 | u | y | n |  | never | 0 | 0 | 0 |
| Aus | y | y | 53 | 14 | y | y | n |  | never | 0 | 0 | 0 |
| Aus | y | u | 26 | 13 | u | u | n |  | never | 0 | 0 | 0 |
| Aus | n | y | 67 | 7 | u | y | n |  | never | 0 | 0 | 0 |
| NZ | y | n | 62 | 6 | u | u | n |  | never | 0 | 0 | 0 |
| Aus | y | y | 44 | 5 | n | n | n |  | never | 0 | 0 | 0 |
| Aus | n | y | 49 | 5 | n | y | n |  | never | 0 | 0 | 0 |
| NZ | y | y | 51 | multiple | u | u | n |  | unknown | 22 | 22 | 20 |
